# A Comparison of Machine Learning and Deep Learning Techniques for Activity Recognition using Mobile Devices

**DOI:** 10.3390/s19030521

**Published:** 2019-01-26

**Authors:** Alejandro Baldominos, Alejandro Cervantes, Yago Saez, Pedro Isasi

**Affiliations:** Department of Computer Science, University Carlos III of Madrid, 28911 Leganés, Madrid, Spain; acervant@inf.uc3m.es (A.C.); yago.saez@uc3m.es (Y.S.); isasi@ia.uc3m.es (P.I.)

**Keywords:** deep learning, activity recognition, mHealth, safe environment, active aging, ambient-assisted living

## Abstract

We have compared the performance of different machine learning techniques for human activity recognition. Experiments were made using a benchmark dataset where each subject wore a device in the pocket and another on the wrist. The dataset comprises thirteen activities, including physical activities, common postures, working activities and leisure activities. We apply a methodology known as the activity recognition chain, a sequence of steps involving preprocessing, segmentation, feature extraction and classification for traditional machine learning methods; we also tested convolutional deep learning networks that operate on raw data instead of using computed features. Results show that combination of two sensors does not necessarily result in an improved accuracy. We have determined that best results are obtained by the extremely randomized trees approach, operating on precomputed features and on data obtained from the wrist sensor. Deep learning architectures did not produce competitive results with the tested architecture.

## 1. Introduction

Human activity recognition is a field of study with significant research interest, mostly due to a couple of reasons. First, a system involved in what the user is doing can provide a more customized experience and improved interaction. For example, if a mobile operating system (such as Android or iOS) detects that the user is focused on working, then it can automatically reduce the frequency of non-critical notifications, such as those from messaging apps. Health apps could track when users are exercising or brushing their teeth, congratulating the user in certain cases. Also, an app for quitting smoking could detect when the user is smoking and tell them off.

The second reason is about technology: the pervasiveness of sensors in wearable devices has enabled researchers to study how to achieve successful human activity recognition using this kind of technology, whose use by users is growing year after year. In this sense, nowadays most people carry a smartphone in their pockets or purses for long periods of time in their daily lives, and some people are adopting so-called smartwatches on their wrists. These devices are equipped with sensors such as accelerometers, gyroscopes, magnetometers or even heart rate monitors. The data gathered by these sensors can be used to learn about the activities performed by the user when carrying these devices.

In this paper, we will explore human activity recognition using two smart devices: one worn in the pocket and the other on the wrist. We will tackle this problem from a systematic machine learning approach: we want to study how different machine learning techniques behave when classifying physical activity. For this study, we will compare different classical machine learning techniques as well as convolutional neural networks (CNNs), which are a technique comprised within the recent field known as Deep Learning. We will also explore the impact of different data preprocessing and segmentation techniques.

This paper is structured as follows: First, Section 2.1 describes the problem of human activity recognition, providing a taxonomy for the different tasks within this research field and surveying the current state of the art. Then, Section 2.1.1 and Section 2.1.7 describe classical machine learning (ML) techniques and CNNs respectively, explaining how they will be applied in the paper. Later, Section 2.2 explains the methodological approach of this work, describing first the dataset that we will use in this work and later the different stages for transforming, segmenting and extracting features from the data, until classification is performed. The classification performance is explored in Section 3, and the results obtained are discussed in further depth. Finally, Section 5 provides some conclusive remarks as well as future lines of work.

## 2. Materials and Methods

### 2.1. Learning Techniques for Human Activity Recognition

Human activity recognition is a field of study which has gained significant attention in recent years due to the pervasiveness of sensors, which are now available in smartphones and wearable devices (such as smart watches or smart wristbands).

**Figure 1:**
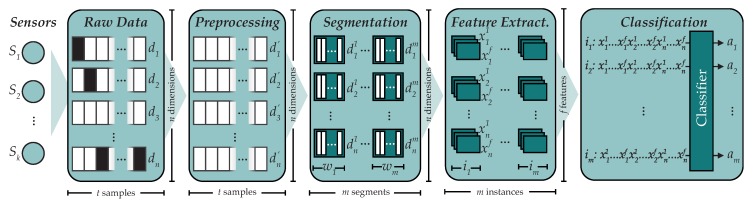
The five steps contained in the activity recognition chain: acquisition, preprocessing, segmentation, feature extraction and classification (extracted from Ref. [2]).

Activity recognition can be seen as a classification problem, a common type of problem found in supervised learning. In 2014, Bulling et al. [1] studied this problem and proposed the *activity recognition chain* (ARC), a sequence of steps in order to build human activity recognition systems (see Figure 1), to which we will adhere in this paper. Bulling et al. also taxonomized these kinds of systems according to different criteria:

- According to its execution mode, the system can be offline or online. In the latter case, the system is able to perform activity recognition in real time.
- According to its generalization ability, the system can be user-independent or user-specific. In the former case, the system should be able to recognize the activity even for those users who are using the system for the first time.
- According to the type of recognition, the system can process continuous streams of data or isolated data where the offset of each activity is previously known.
- According to the type of activities, they can be periodic, sporadic or static.
- According to the system model, it can be stateless or stateful. In the latter case, the system not only is aware of the sensors data but also considers a model of the environment.

In this work, we will consider a user-independent, continuous, stateless system. It could work either offline or online, and will mostly recognize periodic and static activities.

We must note there are limitations to the generalization ability of the selected approach. Due to the characteristics of the available data, we cannot be certain of how the results would differ if tested on a different group of users. However, we are not so interested in actual success percentages but in the comparison on different techniques in equal conditions.

Another important matter, raised in Ref. [3], is the assumption regarding environmental conditions during the construction of the system. In our case, limitations on available data suggested the use of a trained approach, that is, we are training all systems assuming the same conditions both for training data and test data. This may or may not be the applicable case to a given real case. A stateful system would be more adequate in order to take into account environmental changes. However, information on the data acquisition environment was not available to us. We are thus, working on the assumption that personal sensors are designed to reduce environmental influences, and that may limit the significance of this element in this particular case.

Human activity recognition is a field which can be closely related to mHealth (mobile health), since in most cases, the activities to be recognized have a significant impact in the subject’s health. For example, in this work we will focus, among others, on certain physical activities (such as running, walking or jogging) and undesirable activities such as smoking. A system able to track the amount of physical exercise performed by the user could raise self-awareness and give proper credit for this task, an essential requirement to promote physical activity [4]. Also, when the user carries out an undesirable activity, such as smoking, the system could raise an alert. The relationship between mHealth and activity recognition has been studied by Dobkin and Dorsch [5].

We will use sensors located in smartphones and wearables instead of ad-hoc sensing devices placed across the subjects’ bodies. This is essential to transfer this technology to the society, since most users are already equipped with this type of device, and would not be required to hold additional hardware. Some applications of mobile activity recognition have been addressed and proposed by Lockhart et al. [6], including some closely related to mHealth, such as fitness tracking or health monitoring.

As we stated before, an important aspect of human activity recognition applied to mHealth is recognizing physical activities (exercising). One of the most relevant datasets for physical activity is PAMAP (Physical Activity Monitoring in the Ageing Population), which was introduced by Reiss and Stricker [7,8]. This dataset was used by Saez et al. [2] in order to compare the performance of different machine learning and deep learning classifiers for cross-person classification, and later Baldominos et al. [9] used it for optimizing the feature extraction stage of the activity recognition chain.

In 2015, Shoaib et al. published a survey of online activity recognition systems using data from mobile phones [10]. Since then, different machine learning techniques have been applied to the recognition of activities using mobile and wearable devices, such as naive Bayes, *k*-NN and decision trees [11], or the Ameva algorithm, the latter also supporting fall detection in the aging population [12].

In the last couple of years, deep learning has started to become a standard for human activity recognition. An example of an early application is that by Ordoñez and Roggen [13], where they used a combination of CNNs along with Long short-term memory (LSTM) layers in order to carry out activity recognition using the OPPORTUNITY dataset, which uses opportunistically discovered sensors along the subjects’ bodies and the environment [14]. Later, some applications have arisen of deep and convolutional neural networks for mobile activity recognition. For example, Ordoñez and Roggen [15] studied the effect of transfer learning between different domains, modalities and location. Inoue et al. [16] used deep recurrent neural networks to improve the recognition accuracy in 20–30 percentage points when compared to classical approaches, increasing the recognition throughput. Finally, Münzner et al. [17] have explored the use of CNNs for multimodal activity recognition using sensor fusion techniques.

#### 2.1.1. Classical Machine Learning Techniques

In this paper, we will test the performance of different classical machine learning techniques for human activity recognition.

In this section, we will briefly describe the different techniques that will be used. These techniques will be taxonomized according to the following categories: linear models, probabilistic models, geometric models, neural networks and ensembles.

In this paper, we have used the implementation of the following algorithms provided in the *scikit-learn* [18] library for Python. We have chosen to adhere to this library because it enables fast prototyping of machine learning programs, eases the comparison of different techniques and allows the replication of the experiments.

#### 2.1.2. Linear Models

Linear models are those that make a decision on the output based on a linear combination of the input. In this paper, we will use logistic regression, which is a widely used classification technique.

Given an input vector $x\in R^{n}$, the logistic regression parameters will comprise vector of weights $w\in R^{n}$ and a bias value b∈R. The output of the logistic regression for such input will be computed as:

(1) $$\hat{y}=\sigma \left(w^{⊤}x+b\right)$$

where $\sigma \left(z\right)$ is the logistic (or sigmoid) function:

(2) $$\sigma \left(z\right)=\frac{1}{1+e^{-z}}$$

Because $\hat{y}$ will be a real number in the range [0,1], binary classification can be achieved by considering that the model will output the class 0 if $\hat{y}<0.5$ and the class 1 otherwise.

In this paper, we will use a one-vs-rest approach for multinomial classification, using the LIBLINEAR optimizer [19] for learning the model parameters and applying L2 regularization.

#### 2.1.3. Probabilistic Models

Probabilistic models perform supervised learning by inferring an underlying probability distribution of data. The best well-known method is naive Bayes, which works by applying the Bayes theorem assuming strong independence among features. With this assumption, the conditional probability of *y* given the input vector $x\in R^{n}$ is:

(3) $$P\left(y|x_{1},\cdots ,x_{n}\right)=\frac{P\left(y\right)\prod_{i=1}^{n}P\left(x_{i}|y\right)}{P\left(x_{1},\cdots ,x_{n}\right)}$$

Given the previous formula, we can estimate the most likely class as follows:

(4) $$\hat{y}=\underset{y}{arg max}P\left(y\right)\prod_{i=1}^{n}P\left(x_{i}|y\right)$$

In this paper, we will use Gaussian naive Bayes, where the likelihood of the features is assumed to be Gaussian:

(5) $$P\left(x_{i}|y\right)=\frac{1}{\sqrt{2\pi \sigma_{y}^{2}}}\exp\left(-\frac{{\left(x_{i}-\mu_{i}\right)}^{2}}{2\sigma_{y}^{2}}\right)$$

where $\sigma_{y}$ and $\mu_{y}$ are estimated using maximum likelihood [20].

#### 2.1.4. Geometric Models

Geometric models assume that data with similar features can be grouped together, and does so based on the geometric properties of the data. One of the simplest yet most widely used geometric models for classification is *k*-nearest neighbors (*k*-NN) [21]. Interestingly, this algorithm does not build an explicit model, but rather perform *instance-based learning*: new instances are classified by comparing them with the whole training set.

In this paper, we have used *k*-NN with an Euclidean metric, where the distance between two instances $x^{a}$ and $x^{b}$ is computed as follows:

(6) $$d\left(x^{a},x^{b}\right)=\sqrt{\sum_{i=1}^{n}{\left(x_{i}^{a}-x_{i}^{b}\right)}^{2}}$$

To classify an instance, the *k* closest instances are chosen and the class is decided via majority voting. As a result, *k*-NN determines Voronoi regions in the multidimensional hyperspace in order to classify new instances. After a preliminary analysis, we have determined to set k=5, letting features uniformly weighted.

#### 2.1.5. Neural Networks

**Figure 2:**
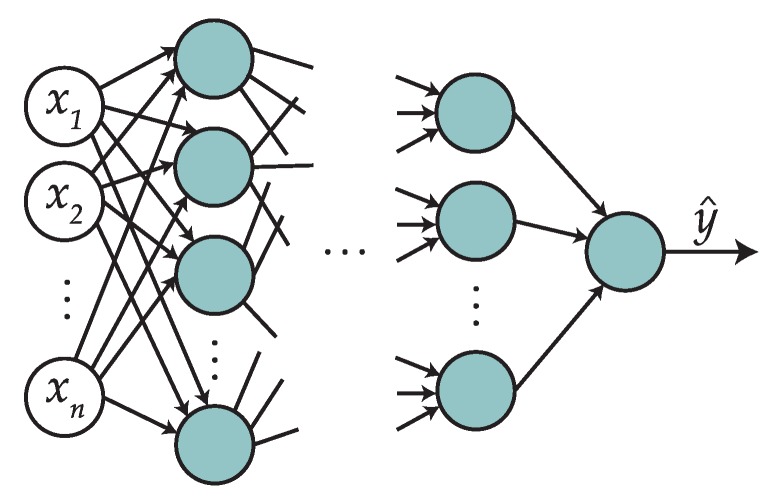
Basic topology of a multi-layer perceptron.

Neural networks are techniques that in a sense try to mimic the behavior of the human brain. In this paper, we will use the multi-layer perceptron (MLP), which is a very commonly used implementation of a feed-forward network that works as a universal function approximator. The basic topology of an MLP is shown in Figure 2.

The first layer (in white) is known as an input layer, and will store the values of the vector $x\in R^{n}$, the feature vector for a given instance. The following layers, except for the last one, are known as hidden layers. We consider the MLP to have *L* layers (L−1 hidden layers and one output layer), where the *l*-th hidden layer has $n_{l}$ units (or neurons).

During classification, data is going forward through the different layers. A layer *l* is characterized by two sets of parameters: a matrix of weights $W^{l}\in R^{n_{l}\times n_{l-1}}$ and a vector of biases $b^{l}\in R^{n_{l}}$.

The activation vector of neurons in layer *l* is computed as follows:

(7) $$a^{l}=g\left(W^{l}a^{l-1}+b^{l}\right)$$

where g(·) is the activation function of neurons in layer *l*. In this paper, we will use the ReLU (Rectified Linear Unit) activation function for the hidden layers, which is a non-linear function defined by the next equation:

(8) g(x)=max(0,x)

ReLU has been proved a useful activation function since it prevents the effect of vanishing gradient, thus, accelerating the learning of the parameters during backpropagation [22].

The last layer is known as the output layer. In the case of binary classification, the output layer has one output unit, and computes the output as follows:

(9) $$y=\sigma (W^{L}a^{L-1}+b^{L})$$

In this case, we will not use the ReLU function, but a logistic function σ (see Equation (2)) so that the values are normalized in the [0,1] range. In the case of multinomial classification, the MLP will have more than one output layer.

In this paper, we have used a MLP with two hidden layers, with 200 and 50 units respectively. The network will be trained using the Adam optimizer [23] with the suggested values for $\beta_{1}$, $\beta_{2}$ and ϵ, and a learning rate of α=0.0001. The network will be trained for 2000 epochs.

#### 2.1.6. Ensembles

Ensembles combine different machine learning classifiers in order to obtain an overall improved model. The idea behind ensembles is that each model is specialized in a certain set of instances, and when working together they often perform majority voting in order to decide which class should be assigned to a certain instance.

While ensembles can be built out of any machine learning model, the most common approaches involve ensembles of decision trees. A decision tree is a structure that makes questions about the instance features in each level of the tree, descending through the branches until reaching a leaf, which contains the preferred class for that instance. A commonly used implementation of a decision tree is Quinlan’s C4.5 [24].

In this paper, we will compare two different implementations for building ensembles of decision trees: random forests [25] and extremely randomized trees (ET) [26]. In both cases, each of the trees is trained using a random sample of the training data. We have configured both ensembles to train 20 decision trees using Gini impurity.

#### 2.1.7. Convolutional Neural Networks

Convolutional neural networks [27,28] are a specific type of deep learning networks that are being applied to many complex problems with significant success. A convolutional network is composed of two differentiated parts: a feature learner and a classifier. The feature learner is made of a convolutional layer, most often placed sequentially, where each layer can process the output of the previous one in order to generate feature maps, which after learning, will turn into useful features extracted directly from the data without the need of manual feature engineering. Between these convolutional layers, pooling layers can be optionally found to decrease the feature maps size using downsampling. Finally, the classifier is often implemented as a MLP or conversely, a recurrent neural network.

The main strength of CNNs is that they are able to automatically extract relevant features that can be useful for classification, without the need of human experts deciding on which features are the most convenient. These features are automatically introduced to the classifier to generate an output. Interestingly, during the backpropagation process both the parameters of the classifier and the feature learner are trained, therefore providing a global optimization process (which contrasts the classical feature engineering process, when human intervention would be needed if the first set of features did not reach the expected quality). Another important aspect of CNNs is the introduction of pooling layers. Besides reducing dimensionality of feature maps, they avoid mapping a specific feature to an absolute “position” in the network connections, effectively providing invariance to translations. This was found to be quite useful in computer vision applications, where the network learns to recognize specific characteristics such as a given pattern of lines or colors, in any part of an image provided as a pixel matrix regardless of its position.

**Figure 3:**
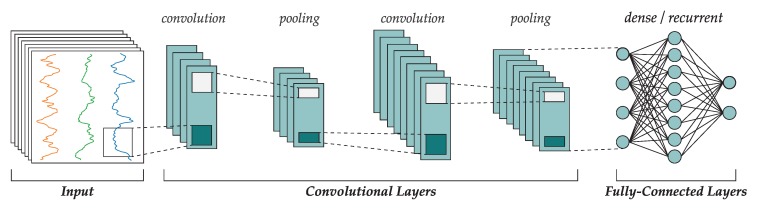
Common topology of a CNN (extracted from Ref. [29]). For signal processing, input vectors are sequences of values for all the signals, concatenated for a fixed-size window of time.

Figure 3 shows the topology of a typical CNN where the input raw data passes through two sets of convolutional and pooling layers. The resulting data is a set of calculated feature maps that are passed to the fully connected layers that are indeed a MLP or recurrent network that is trained in a conventional way using gradient descent. In signal processing applications such as this work, each input vector the concatenated values for the sensor measure over a window of time of length *W*.

Convolutional layers are composed of a series of kernels (also known as filters) that process their inputs by performing a convolution operation. These elements work on multidimensional arrays (tensors), which in signal processing would often be one-dimensional while in computer vision they would be two-dimensional.

**Figure 4:**
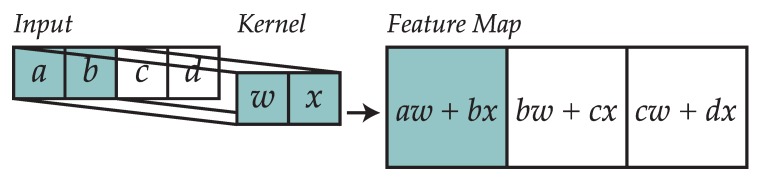
Example of how a kernel is used to convolve the input (extracted from Ref. [29]).

Convolution of the input tensor with the kernel produces the kernel output as shown in Figure 4. The convolution operator for a one-dimensional vector is shown in Equation (10). The process of convolution, followed by pooling, enables the network to search for correlations among consecutive groups of input values that can be present in different parts of the analyzed window.

(10) $$S\left(i\right)=\left(K*X\right)\left(i\right)=\sum_{m}X\left(i-m\right)K\left(m\right)$$

As a result, for an input $X\in R^{i}$ and a kernel $K\in R^{m}$, the resulting tensor will be shaped $A\;\in \;R^{\left(i-m+1\right)}$.

After computing feature maps, an activation function can be applied to every element in the output tensor. In our case, we will apply a ReLU function in order to compute a non-linear transformation of the output.

Each pooling layer reduces the dimension of its input, typically using the technique of *max-pooling*. In this technique a group of input data is replaced by the maximum value of the group.

After the convolutional layers have extracted relevant features from the input data, the resulting tensor will be flattened into a vector. Then, this vector can be introduced to a trainable learning, which will often be a fully connected neural network.

Whereas a common approach could be to use a MLP, we have decided to use a recurrent neural network. However, instead of the classical recurrent approach, which is harder to train, we have preferred to introduce LSTM cells [30].

**Figure 5:**
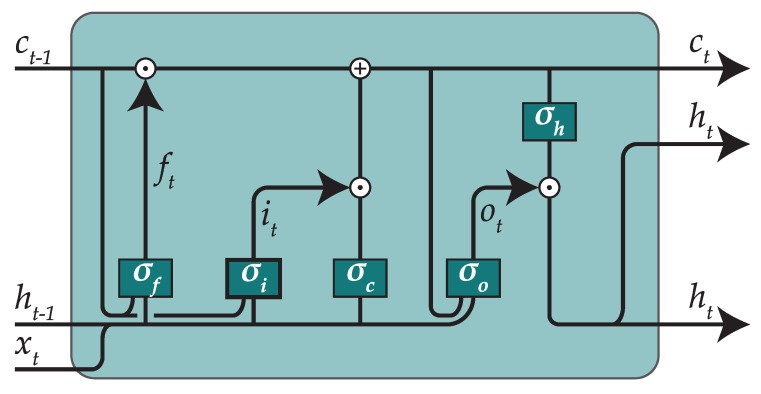
Typical structure of a LSTM cell.

The architecture of a LSTM cell with peephole connections is shown in Figure 5. In LSTM cells, $c_{t}$ is called the “cell state”. A cell contains three gates that control the cell state by adding or removing information. The first gate is the “forget gate” and decides which information will be forgotten from $c_{t-1}$, the second gate is the “input gate” and decides which new information will be included into $c_{t}$, and the last gate is the “output gate” and decides which information will constitute the output. The math behind a LSTM cell is shown in the next equations, where ⊙ stands for an element-wise product operator:

$$\begin{array}{cc} f_{t} & =\sigma_{f}\left(x_{t}W_{xf}+h_{t-1}W_{hf}+w_{cf}⊙c_{t-1}+b_{f}\right)\\ i_{t} & =\sigma_{i}\left(x_{t}W_{xi}+h_{t-1}W_{hi}+w_{ci}⊙c_{t-1}+b_{i}\right)\\ c_{t} & =f_{t}⊙c_{t-1}+i_{t}⊙\sigma_{c}\left(x_{t}W_{xc}+h_{t-1}W_{hc}+b_{c}\right)\\ o_{t} & =\sigma_{o}\left(x_{t}W_{xo}+h_{t-1}W_{ho}+w_{co}⊙c_{t}+b_{o}\right)\\ h_{t} & =o_{t}⊙\sigma_{h}\left(c_{t}\right) \end{array}$$

The first of these gates, $\sigma_{f}$ is the “forget gate” which will look at the input and decide which information will be forgotten from $c_{t-1}$. In particular, $f_{t}$ will be a vector of real values in the interval [0,1], that will be multiplied element-wise by $c_{t-1}$. A 0 means that the corresponding information in $c_{t-1}$ will be ignored, whereas a 1 would leave that information unaltered.

The second gate, $\sigma_{i}$ is called the “input gate”, and decides which new information will be included into the cell state. In particular, $i_{t}$ will be a real vector with the same format than $f_{t}$, and a function $\sigma_{c}$ will transform the input data. Later, the transformed data and $f_{t}$ will be multiplied element-wise, and the result will be summed up with $c_{t-1}$, resulting in $c_{t}$.

The final gate, $\sigma_{o}$, is called the “output gate” and will decide which information will constitute the output. Here, $o_{t}$ will be a vector similar to $f_{t}$. However, it will not be used to modify the cell state; instead, it will be applied over a transformed version of the cell state in order to be returned as output.

By using LSTM cells, we will be able to learn temporal components of the data, which can be useful since we will be dealing with time series.

Again, we have chosen to apply ReLU as the activation function for the fully connected layers. Also, we will introduce *dropout*, a technique that removes a random set of connections during training which has been proven successful [31] as a form of regularization to avoid overfitting.

In order to implement the convolutional neural network, we have used the Theano framework [32] along with the Lasagne library. We have chosen to use these tools since they simplify the construction of the CNN topology and speeds up the training process. Also, we can share the code with the scientific community to enable them to replicate the experiments.

For designing the CNN topology, we have performed a prior analysis to determine suitable hyperparameters. In the end, we have decided to include three convolutional layers with 64 kernels each, with a filter size of 10 and a ReLU activation function. A max-pooling layer of size 2 was included after each convolutional layer. Also, we have included two LSTM layers with 128 units each and ReLU activation function, and dropout of 50%. The output layer would implement a softmax function: by doing so, all the output values sum up to 1, and they can be seen as the probability distribution of the instance belonging to any of the classes.

For training the network we have used the RMSProp optimizer [33] with a learning rate of α=0.001 and the suggested hyperparameters: ρ=0.9 and $\epsilon =10^{-6}$.

### 2.2. Methodology

A sequence of steps for working on human activity recognition problems, namely the *activity recognition chain* (ARC) was introduced by Bulling et al. [1]. The steps involved in the ARC were previously shown in Figure 1 and include: data acquisition, data preprocessing, segmentation, feature extraction and classification.

In this paper, we will adhere to the activity recognition chain when working on classification using classical machine learning techniques. When using CNNs instead, we will use a slightly different approach as a part of an end-to-end deep learning solution, which does not require the feature extraction stage.

In the current section, we will thoroughly describe how each of these stages are applied in this work.

#### 2.2.1. Data

For this paper, we have decided not to perform data acquisition on our own, but rather use a publicly available dataset. In particular, we have chosen to use the data provided by Shoaib et al. [11,34]. This dataset contains labeled instances for activity gathered using two mobile devices and is quite recent, as it has been released in 2017. By using a public dataset, we enable reproducibility of the results of this paper. Data can be downloaded from the Pervasive Systems group page of University of Twente [11].

This dataset contains data for thirteen different human activities, including physical activities (walking, jogging, biking, going upstairs and going downstairs), common postures (standing, sitting), working activities (typing, writing), and leisure activities (talking, eating, drinking coffee and smoking).

The data was collected from ten healthy male participants aged 23–35 years. The physical activities and common postures were performed by all subjects. Working activities and leisure activities were performed by seven out of the ten subjects, with the sole exception of smoking, which was performed by six participants (only six of them were smokers, and non-smokers were not required to smoke for the data collection stage). A total of 30 min of data is available for each activity, with an equal amount of data from each participant. Thus, the dataset involves 390 min of data.

The participants were asked to carry two mobile phones Samsung Galaxy S2, one in their right pocket and the other in the right wrist. An exception was made since one subject was left-handed: in that case, he was allowed to carry the device in his left wrist, and data was post-processed accordingly to make it consistent with the remaining data. The orientation of mobile phones was portrait with their screen pointing towards the body. The wrist-worn smartphone is used as a proxy for an actual smartwatch device, having equivalent sensors. Data is sampled from the accelerometer, the gyroscope and the magnetometer at a frequency of 50 Hz using an Android app specifically designed for that purpose [34]. Additionally, data from a virtual linear accelerometer is also included in the dataset, by removing acceleration due to gravity from the accelerometer data. We performed no additional data calibration besides the one already in the raw data provided in the publicly available dataset. The resulting raw data sample vector has 12 components, detailed in Table 1.

**Figure 6:**
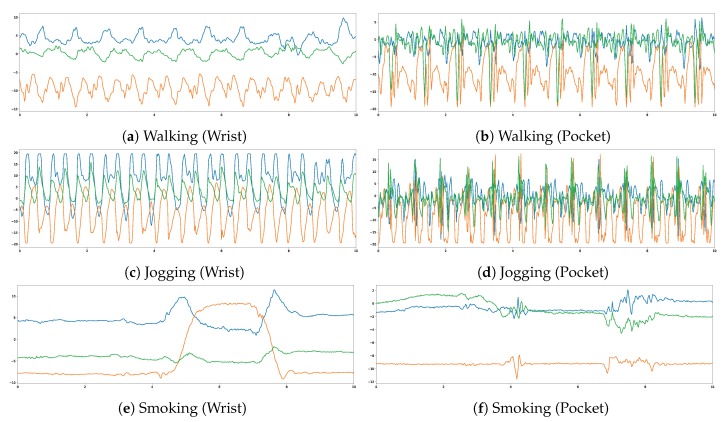
Motion captured by the accelerometer (in $ms^{-2}$) located in the wrist (**left**) and the pocket (**right**), for ten seconds of walking, jogging and smoking. Blue is the *x* component, orange is the *y* component, and green is the *z* component of the measure.

Some examples of the data available in the dataset are shown in Figure 6. Each plot shows the value of the three dimensions of the accelerometer (blue is *x*, orange is *y*, green is *z*) for a certain activity. Plots in the left side belong to the accelerometer located in the wrist, while those in the right refer to the accelerometer located in the pocket. Interesting trends can be seen with just these few examples, and we will now mention a few. Walking and jogging have a very similar effect in the accelerometer in the pocket, the main difference being that when jogging, both the magnitude and frequency of the motion are larger. However, walking and jogging are quite different when the motion is captured in the wrist: walking barely has any motion, but jogging does. The activity of smoking is rather interesting: most of the time, the plot shows barely any movement. However, we can see how between seconds 4 and 8 the *y* dimension of the accelerometer located in the wrist experiences a large motion, which would reflect the fact that the subject carries the hand to the mouth in order to aspire smoke. This action takes about four seconds, and then the subject comes back to the original position. Interestingly, about the 4th and the 8th second, there is a small impact on the accelerometer in the pocket, which could happen if the subject be moving the hand holding the cigarette near the smartphone located in the right pocket.

#### 2.2.2. Preprocessing

The data has been used as provided by Shoaib et al., and has not been submitted to further preprocessing. Original data is already synchronized and cleaned, and we have used it out-of-the-box. However, we have omitted the timestamp, since it is not a really useful feature for learning the activity performed.

#### 2.2.3. Segmentation

**Figure 7:**
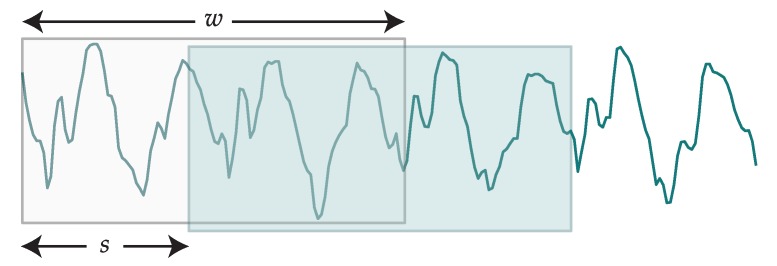
Conceptualization of a sliding window of size *w* and step *s* applied over one axis.

For the segmentation stage, we have created segments by moving a sliding window across each dimension of the data. The process is conceptually depicted in Figure 7. The sliding window will have a fixed size of *w* samples and a step of *s* samples. Every time the window slides over the data, a new segment is created with the samples contained in the window. The larger the step, the less data we will have after feature extraction; on the other hand, very small steps can lead to unmanageable volumes of data. Windows are guaranteed to comprise only instances belonging to the same activity.

Each input vector $x_{i}$ is obtained from the sequence of training data for an activity $T_{a}$ using Equation (11), where we address the elements in *T* using the slice notation typical in arrays.

(11) $$x_{i}=T_{a}\left[x_{s_{start}},x_{s_{start}+w-1}\right]\;s_{start}=s*i$$

#### 2.2.4. Feature Extraction

In order to introduce the data to a classifier, we first need to extract features from the raw data. This step will only be performed when classification is done via classical machine learning techniques. When using convolutional neural networks, this step is ignored and the segments obtained in the previous stage are introduced directly to the first convolutional layer.

In this stage, we will manually engineer features that enable the classifiers to learn the class from the data. First, it should be noted that the data can be seen as a time series. When dealing with classification of temporal series, it is often interesting to transform the series into the frequency domain by computing the discrete Fourier transform (DFT) of the input. In our case, we have computed the fast Fourier transform (FFT) for each dimension in each segment:

(12) $$x_{k}=\sum_{n=0}^{w-1}x_{n}e^{-i2\pi k\frac{n}{w}}\;k=0,1,\cdots ,w-1$$

Then, we have considered only the real coefficients, ignoring the imaginary part of the transformed values. Finally, we have computed the next features for each segment and dimension:

- The mean value of the raw vector.
- The standard deviation of the raw vector.
- The median of the transformed vector.
- The lower quartile of the transformed vector.
- The upper quartile of the transformed vector.
- The skewnwss of the transformed vector.
- The kurtosis of the transformed vector.

We have selected the mean and standard deviation of the non-transformed values because they have been proved useful for activity recognition [35,36]. The statistical values obtained from the transformed input have been chosen because they have been proved useful in previous works [2,35], and have resulted as well of a prior manual stage of sensitivity analysis.

#### 2.2.5. Classification

In order to perform classification, we have divided the data into a training set and a test set. To do so, we have considered the first 70% of the data for each activity as the training set, and the remaining 30% of the data as the test set. Data is not previously shuffled; therefore, the test set contains “future data” with respect to the training set.

The classification is performed with three different datasets: data obtained from the wrist device, data obtained from the pocket device, and both. In the former two cases, the dataset has 12×7=84 features (12 dimensions and 7 features per dimension). In the latter case, the dataset comprises twice the number of features (168), as this dataset results from stacking the other two horizontally.

The classifiers used for learning the activity recognition models have been presented in Section 2.1.1, along with their configuration. Their parameters have been estimated after a prior manual sensitivity analysis. Also, convolutional neural networks will be used for classification, following the description provided in Section 2.1.7. In this case; however, the previous features will not be used, and instead inputs will be one-dimensional vectors formed by the concatenation of the 12 dimensions for the full window: that is, $x\in R^{12\times w}$ for the wrist and pocket datasets, and $x\;\in \;R^{24\times w}$ in the dataset that uses both sensors (wrist and pocket).

### 2.3. Experimental Setup

#### 2.3.1. Classical Machine Learning Methods

The experiments have been executed in a MacBook Pro laptop with an Intel Core i7 processor running at 3 GHz and 16 GB of DDR3 RAM memory. As for the software, we have used the implementations of machine learning algorithms provided by *scikit-learn* 0.18.1, using *numpy* 1.13.1 and *scipy* 0.19.0 for processing the data in order to extract features.

The machine learning techniques have been described in Section 2.1.1, where some of their configuration was already discussed. For determining the best parameters, we have performed a preliminary sensibility analysis, and concluded that in most cases default parameters work fine, or there are not a significant differences when changing the values. All the relevant parameters of the machine learning algorithms are shown in Table 2.

For the segmentation phase, we have used a sliding window size of W=3000, which is equivalent to 1 min of activity, and a step of S=1500. Reducing the step would provide more training and test data, at the cost of increasing memory requirements and execution time.

Because some techniques may benefit from data normalization, we have decided to normalize the input features by substracting the mean and dividing by the variance. As we will see later, this will have a positive effect in techniques based on numerical computations (MLP, Logistic Regression) and distances (*k*-NN).

Finally, some techniques are stochastic, specifically random forests, extremely randomized trees and the multi-layer perceptron. For this reason, we have executed each classifier 20 times using different random seeds in order to avoid biases and check the robustness of the technique.

#### 2.3.2. Deep Learning Methods

The experiments performed with convolutional neural networks were executed in a workstation comprising 2 Intel Xeon E5-2667 CPUs running at 3.20 GHz, 128 GB of RAM memory, and four NVIDIA TESLA P100 GPUs, plus one Titan Xp GPU.

This workstation has been equipped with the following software packages: *numpy* 1.13.1, *scipy* 0.19.0 and *scikit-learn* 0.18.1. For deep learning we have decided to use the Theano framework [32] in its version *theano* 0.9.0, as well as *lasagne* 0.2.dev1 library for simplifying the creation of the network architecture.

In order to train and evaluate the convolutional neural network using the GPUs, we have installed CUDA Toolkit 8.0, cuDNN 6.0 primitives and *pygpu* 0.6.9.

**Figure 8:**
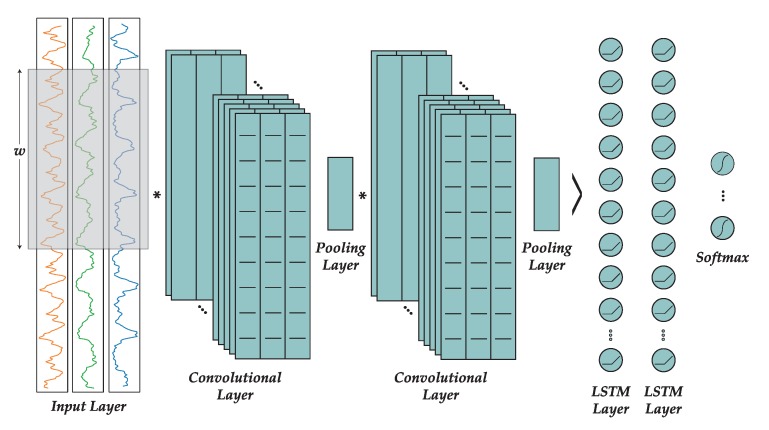
Convolutional neural network architecture.

In Section 2.1.7 we already described the topology of the CNN, decided after manually trying different architectures. This architecture is shown in Figure 8. The network comprises three convolutional layers, each of them with 64 kernels of size 10 and ReLU activation function. After every convolutional layer there is a pooling layer of size 2 in order to halve the size of the feature maps. Later, the output feature maps are flattened and introduced into a recurrent network consisting on two layers with 128 LSTM cells each, and ReLU activation function, with a 50% of dropout regularization. Finally, the output is passed through a softmax function to normalize the values between 0 and 1 in order to complete the classification process.

We have chosen a window length of W=3000, as it was the value tested with classical machine learning techniques. However, in this case we used a step of S=100, since deep learning techniques benefit from the availability of large quantities of data. For the wrist and pocket datasets, minibatches of B=75 windows were introduced to the network, whereas for the combined dataset the batch size was of B=35. These values were as large as possible to avoid too much stochasticity during training, while allowing the data to fit in memory.

For the learning rate, we established an initial value of α=0.1, which was adjusted over time in order to reduce it by 0.95 every time the train error increased from one epoch to the next. By doing so, we can achieve a fast optimization at the beginning, providing a fine tuning of the network parameters in the end and avoiding the algorithm from diverging.

## 3. Results

In this section we will highlight the results attained using different machine learning techniques, including convolutional neural networks.

As stated before, the empirical work performed in this paper is twofold. The first approach carefully follows the steps of the activity recognition chain in order to generate a new dataset with the extracted features that will be introduced to different classical supervised learning techniques in order to perform classification. The second approach introduces the segmented data into a convolutional neural network directly, omitting the feature extraction stage. In this section, we will describe the experimental setup and the results for each of these approaches separately.

### 3.1. Classical Machine Learning

Now we will proceed to discuss the experimental setup and the results obtained using classical supervised learning techniques.

First, we will compare the performance of the different classifiers for each dataset, i.e., when activity recognition is performed using the data acquired from the smartphone located (a) at the wrist, (b) at the pocket, or (c) both of them.

**Figure 9:**
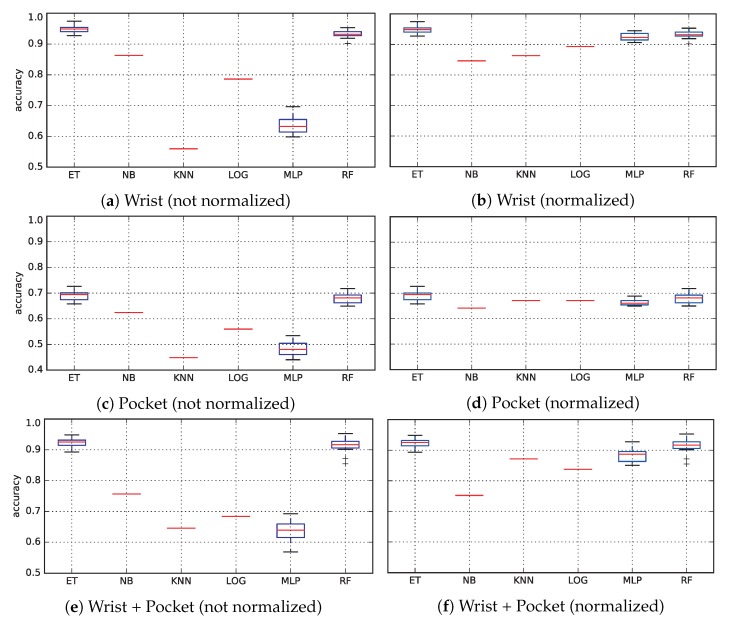
Accuracy for the six classical classifiers for the three datasets, both for not normalized data (**left**) and for normalized data (**right**).

Figure 9 shows a boxplot for each dataset. Results in the left column have been obtained using data as returned from the feature extraction stage. Meanwhile, results in the right have been submitted to an additional normalization process; therefore, both training and test data has 0-mean and unitary variance. The reason why some values are only represented by a red line instead of a box is that these three methods (naive Bayes, logistic regression and *k*-NN) has a deterministic implementation and do not rely on any random variable.

It can be seen how normalization significantly improves numerical methods (logistic regression and multi-layer perceptron) and geometric techniques (*k*-NN). On the other hand, Naive Bayes and ensembles of decision trees are not really affected by this process.

Also, it seems clear that the smartphone located at the pocket is insufficient for successfully tackling activity recognition, since the accuracy never exceeds 70%, whereas the smartphone at the wrist achieves accuracies over 96%. Interestingly, the dataset combining both smartphones does not improve the results when compared to the wrist data. In fact, accuracy achieved by the top-performing models (random forests, extremely randomized trees and the multi-layer perceptron) are slightly worst when both datasets are combined. This could happen due to the larger dimensionality of data: the last dataset has twice as much features as each of the other datasets.

The best results are consistently provided by the ensembles of decision trees (with a slightly better performance of extremely randomized trees over random forests), closely followed by the MLP when the data is previously normalized. Other techniques are able to compete with these techniques in some datasets, but that performance is not consistent over all the datasets.

The accuracy and F1 score for each classifier are shown in Table 3 for the wrist dataset, Table 4 for the pocket dataset, and Table 5 for the combined dataset. For stochastic classifiers, both the average and the maximum values are shown in the tables. In all cases, extremely randomized trees attains both the best average accuracy and average F1 score; and with the exception of the combined dataset, it also obtains the best maximum values for these metrics.

Finally, we will study how accuracy and F1 score are affected by the window size. To do so, we have trained and evaluated 20 models of extremely randomized trees for all window sizes between 1 and 60 s.

**Figure 10:**
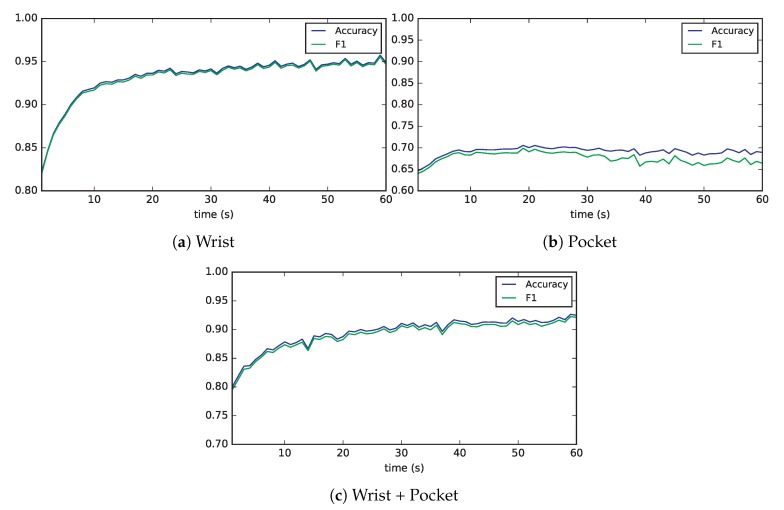
Evolution of accuracy and F1 score as the window size increases for the extremely randomized trees model.

Figure 10 plots the average accuracy and F1 of these 20 executions against the window size. We can see how, for the wrist and combined datasets both metrics are highly correlated and show a logarithmic trend of growth, asymptotically bounded as the window size approaches 60 s. In the case of the pocket dataset, both metrics are also highly correlated but there is a larger gap among them. The evolution is much more flat in this dataset, almost linear for the accuracy, and the average F1 score even slightly decreases as the window grows in size.

### 3.2. Deep Learning

Next, we are going to describe the results obtained using convolutional neural networks.

**Figure 11:**
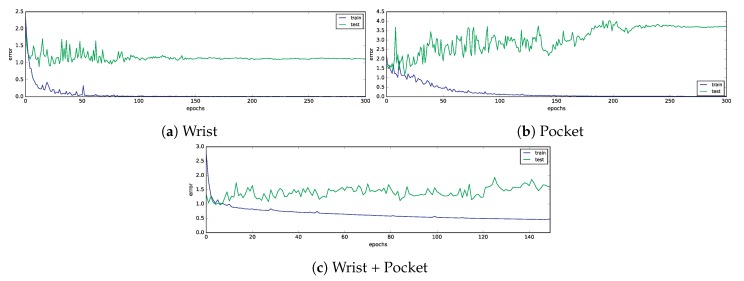
Evolution of training and test error along the training epochs for the deep learning classifier.

The training process of the convolutional neural networks has run for 300 epochs at the wrist and pocket datasets and 150 epochs for the combined dataset. In the former case, each epoch required an average of 282.9 s to complete, whereas in the former case each epoch took in average 620.35 s. The evolution of the train and test error for all the datasets is plotted in Figure 11. We can see how in all cases the training error improves over time. In some cases, training error may increase from one epoch to the next, due to two reasons: the use of microbatching instead of the whole training set and the setting of a large learning rate. The second cause is solved by adjusting this hyperparameters during the training process, thus, guaranteeing convergence as the number of epochs increase.

In the case of the test error, its evolution is significantly more noisy, yet it eventually converges in the wrist and pocket datasets. It does not converge on the combined dataset, but that might be fixed by running the training process over a larger number of epochs. However, the only dataset showing an improvement when compared to the first epochs is the wrist dataset; whereas in the pocket dataset error seems to have an increasing trend over time. It is noticeable that there is a large difference between the training error and the test error. This could be due to overfitting, and partially solved by using larger amounts of data or including additional regularization techniques; however, there are not guarantees that this issue be solved.

**Figure 12:**
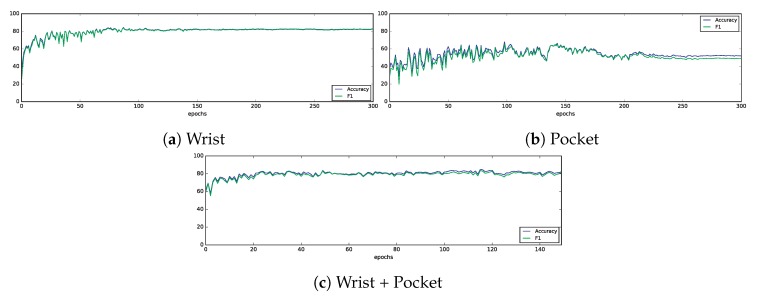
Evolution of test accuracy and F1 score along the training epochs.

The evolution of test accuracy and F1 score are shown in Figure 12. In all cases, we can see how both metrics converge in the last epochs. The wrist dataset shows a clear pattern of improvement, with an initial accuracy of about 20% which is improved in the last epochs to values close to 80%. This effect can also be seen, yet more subtly, in the combined dataset. On the other hand, the pocket dataset does not seem to show a good result, with these metrics slightly increasing first and then, at some point, decreasing.

The accuracy and F1 score for the best model attained for each dataset are shown in Table 6. These results are still far from the best models obtained using extremely randomized trees, yet it should be noticed that the topology and parametrization of the CNNs still present some room for improvement, and the approach is still interesting since the task of manual feature engineering is removed from this process.

## 4. Discussions

Additional analysis can be performed on the experimental results. Besides the fact that not every technique has the same degree of success, it is important to consider that they do not have the same variance either. This can be an important issue in deciding which technique is more adequate in terms of robustness.

It is noted that deep learning has been outperforming traditional machine learning techniques in many different domains where feature engineering is complex. However, for this specific problem, given that we removed the step of feature extraction, that is, FFT and statistical operators (see Section 2.2), the deep learning approach did not outperformed traditional machine learning methods. This stresses the fact that, in order to be competitive with classical machine learning methods with an advanced feature engineering, deep learning must include the overload of finding a complex non-linear transformation of raw features adapted to the problem being solved. In cases where transformations are complex, then deep learning might be the most viable approach, but always considering that the computational and data requirements are always higher [37].

**Figure 13:**
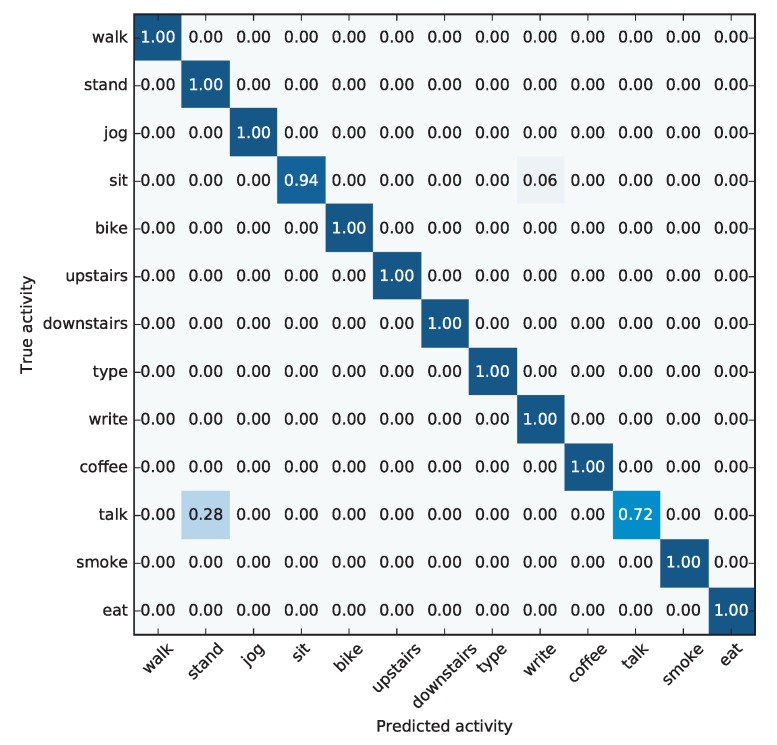
Confusion matrix for the most accurate model.

We have to point out that the confusion matrix analysis is important when working with practical applications. In Figure 13, we show the normalized confusion matrix for this model the best method, extremely randomized trees. This is an useful information, as it motivates the use of further measurements and/or different feature extraction specifically for misclassified activities, if the distinction is necessary for a given application. In our case, adding hearing sensors could solve the confusion among activities “standing” and “talk”.

We can see how that, for ET, the confusion matrix is almost perfect, with many values located in the main diagonal. There are only two exceptions: 28% of talking activities were recognized as standing, and 6% of sitting activities were recognized as writing. These mistakes can have an easy explanation:

- In many cases, it could happen that while people are talking while standing, they move their arms as a form of body language to complement what they are saying. This movement would constitute a source of data which would enable the discrimination between “talking” and “standing” activities. However, it could happen that in some cases subjects remain quiet while talking and do not perform any additional body language, or it is so subtle that it is not captured by the sensors. In this case, discriminating between standing still and talking would be a difficult task, unless additional sensors (such as a microphone or sensors in the face) are used.
- Also, a common difference between writing and sitting is that writing involves moving the hand, and therefore the wrist. In certain cases, if the user is gesticulating while sitting, it could be mistakenly recognized as writing. It is noticeable that the mistake does not occur in the other direction: writing is never confounded with sitting.

The accuracy obtained in this work (97.44%) is very close to that reported by Shoaib et al. [11] (97.53%), yet both of them are not directly comparable as Shoaib et al. used cross-validation for testing the performance. However, their confusion matrix involves a larger number of different mistakes: smoking for standing, talking for eating, typing for sitting, etc.

## 5. Conclusions

In this paper, we have explored the problem of classifying human activity recognition, using a public dataset where the sensors’ data was acquired by using two devices located at the wrist and at the pocket of subjects. Since some activities involve physical exercise, and other activites may carry potential health problems (e.g., smoking), the achievement of successful activity recognition in this domain has potential useful applications in ambient-assited living and healthy aging lifestyle.

To perform activity recognition, we have adhered to the activity recognition chain, a sequence of steps for acquiring, preprocessing, segmenting, extracting features and classifying data. In summary, we have segmented input data by using a sliding window, then computed the fast Fourier transform and finally obtained some relevant features, which have been determined manually. Finally, we have tested six different classifiers using the *scikit-learn* library: extremely randomized trees, random forests, logistic regression, naive Bayes, *k*-nearest neighbors and a multi-layer perceptron. Results have shown that the ensembles of decision trees (i.e., extremely randomized trees and random forests) have achieved the best accuracy in most scenarios.

It is important to remark that, as found in previous works [38], the experimental setup characterizes the data that is going to be delivered to the classifier, and therefore, some configurations can be more precise than others.

Later, we have also tested the application of deep learning techniques, specifically convolutional neural networks. In this case, we have designed an architecture able to automatically learn features from input data without an explicit feature extraction stage. Results are promising, although they do not compete yet with the best models obtained using extremely randomized trees. However, this still is an interesting option with a potential chance to improve its performance after carefully redesigning the network topology and adjusting its hyperparameters.

As a main technical conclusion, as in previous works [2], we observe again that deep learning techniques are not necessarily always the best option when dealing with machine learning and sensory-data models. This is mainly because their performance is highly dependent on the topology and chosen parameters. Another interesting conclusion is that the best accuracy found is 97.44% using only the sensors located at the wrist (F1_Score of 83.96). In this sense, the smartphone placed in the pocket is insufficient to perform accurate activity recognition, and combining both datasets do not improve the results at all. When exploring the confusion matrix, we observe two common mistakes: classifying the activity of talking as standing and the activity of sitting as writing. In certain cases, these mistakes seem to be quite reasonable and have simple explanations. These results would any application to track user’s activity with straightforward applications in care systems, active aging or ambient assisted living. The interaction of these sensors with other devices would complement the ambient intelligence ecosystem.

The source code of this paper has been released in order to enable whole reproducibility of the experiments, and has been uploaded to a public code repository [39].

### Future Work

This work can be easily extended in the future in different ways. First, regarding data acquisition, it would be interesting to use an actual smartwatch or wristband to obtain data from the wrist, instead of a smartphone. In this sense, results should not be affected too much, since both devices usually embody the same type of sensors, yet validating this hypothesis still remains an interesting line of work.

Also, CNNs are showing promising results in this paper, but they still have significant room for improvement. Using specific optimization techniques for determining the best topology and learning hyperparameters would be a good way to reduce overfitting and obtain more competitive results. A possible research line would involve using neuroevolution to obtain better topologies.

Finally, it would be also interesting to explore the performance of cross-person activity recognition. This was not performed in this paper because the dataset we have used has not published independent data coming from different subjects. However, working in the line of improving cross-person classification would enable us to build user-independent recognition systems, which could be potentially used by users out-of-the-box, even when the system has not recorded any previous data from that user.

## Figures and Tables

**Table 1:** Description of components of raw data.

Sensor	Measure	Components
Accelerometer	Acceleration vector	$\left(A_{x},A_{y},A_{z}\right)$
Linear Accelerator	Linear acceleration vector	$\left(L_{x},L_{y},L_{z}\right)$
Gyroscope	Angular acceleration vector	$\left(W_{x},W_{y},W_{x}\right)$
Magnetometer	Orientation	$\left(O_{x},O_{y},O_{z}\right)$

**Table 2:** Classifiers used in *scikit-learn* along with the values set for their parameters.

Name	Classifier
ET	ensemble.ExtraTreesClassifier (n_estimators=20, criterion=‘gini’, max_depth=30)
NB	naive_bayes.GaussianNB ()
KNN	neighbors.KNeighborsClassifier (k=5)
LOG	linear_model.LogisticRegression (penalty=‘l2’, C=1.0)
MLP	neural_network.MLPClassifier (hidden_layer_sizes=(200,100), max_iter=2000)
RF	ensemble.RandomForestClassifier (n_estimators=20, criterion=‘gini’, max_depth=30)

**Table 3:** Classifiers’ accuracy and F1 (wrist). Boldface indicates the best results.

Classifier	Accuracy (%)		F1 Score (%)	
	Avg.	Max.	Avg.	Max.
ET	**94.87**	**97.44**	**94.68**	**97.39**
NB	84.62		84.44	
KNN	86.32		84.92	
LOG	89.32		88.98	
MLP	92.35	94.44	91.85	94.15
RF	93.25	95.30	93.04	95.17

**Table 4:** Classifiers’ accuracy and F1 (pocket). Boldface indicates the best results.

Classifier	Accuracy (%)		F1 Score (%)	
	Avg.	Max.	Avg.	Max.
ET	**68.93**	**72.65**	**66.41**	**70.91**
NB	64.10		62.35	
KNN	67.09		64.96	
LOG	67.09		65.56	
MLP	66.37	68.80	64.74	67.70
RF	67.99	71.79	65.01	69.76

**Table 5:** Classifiers’ accuracy and F1 (wrist + pocket). Boldface indicates the best results.

Classifier	Accuracy (%)		F1 Score (%)	
	Avg.	Max.	Avg.	Max.
ET	**92.48**	94.87	**92.14**	94.87
NB	75.21		74.22	
KNN	87.18		86.02	
LOG	83.76		82.90	
MLP	88.53	92.74	87.86	92.22
RF	91.50	**95.30**	91.13	**95.23**

**Table 6:** CNNs’ accuracies and F1 scores. Boldface indicates the best results for each score.

Data	Accuracy (%)	F1 Score (%)
Wrist	84.41	**83.96**
Pocket	68.06	65.51
Wrist + Pocket	**84.89**	83.55
